# 

**Published:** 1919-07

**Authors:** 


### ANNOUNCEMENTS

### A REPORT OF FRENCH-BELGIAN RELIEF WORK

Our campaign of raising funds for French and Belgian dentists is now well under way and is meeting with hearty support by the profession. It is evident that American dentists realize that they have an opportunity to be of great assistance to their professional brothers at the time of their greatest need. Contributions to June 1st amount to $1,705.47.

It has been ascertained that adequate equipment for the average French and Belgian dentist can be obtained for $500. The League hopes to secure a contribution from each state of at least this amount, and each dentist established will be designated as a beneficiary of a certain state. He will thus be made accountable, as it were, to the state named, and a periodical report rendered if so desired. In other words, each state may take one or more dentists under its wing, as it were, and through this plan have individual as well as collective interest in this great work. Individual contributions will be grouped so that each donor may be informed of its ultimate use in order to lend a personal touch to our work.

Captain Blake A. Sears, of the Replacement Depot, Dental Corps, St. Hignan, France, states in a recent letter that: "L'Aide Confraternelle is O. K. They are doing a good work and much praise should be given them." This is an organization of French and Belgian dentists through which the League will operate.

Kindly look over your unused equipment and send a list of what you can spare, including materials also, to the office of the President of the League, 131 Allen Street, Buffalo, N. Y. Do not delay, but bear in mind that many instruments now idle and rusting may bring bread and life to our suffering brothers.—J. W. Beach.

### RED CROSS NOTES

American Boys Make Furniture for France.—American school boys are about to execute one of the biggest furniture orders ever placed—30,000 chairs and 10,000 tables. Manufacturing this furniture in school and community manual trainng shops throughout the United States, the members of the Junior Red Cross will present it, through the American Red Cross, to the refugees of northern France. These people are pouring back to their devastated homes at a rate that makes the housing problem involved one of the hardest the French Government and the relief agencies have to solve.

About 100,000 school boy carpenters are expected to work on the job, and it is expected that 15,000 families in France will receive one or more pieces of furniture as the result. With each chair and table will go a message of greeting from the Junior Red Cross, containing an addressed card that will enable the recipients to acknowledge the gift.

The necessity for supplying the refugees with furniture arises from the fact that many are without money with which to buy furnishings, while such as have funds can not be supplied in France because of the lack of materials and the destruction of the means of manufacture.

Keeping Him Fit.—Our responsibilities to our wounded are not yet over. The Red Cross acts as the people's intermediary. Debarkation hospitals in the large cities adjacent to ports are crowded to capacity and base hospitals are continually increasing their facilities to care for the wounded who come in with every ship.

One of the greatest needs that presents itself in hastening the recovery of these boys is proper recreation. Deprived of the natural physical ability to seek relaxation they are dependent for mental stimulation on the pleasures that are brought to them.

The Red Cross has planned out a program of social and physical recreation suited to the needs of these recovering boys, and calculated to encourage that spirit of cheerfulness which is so great a factor in their recovery.

Games and sports have been arranged for under the department of Military Relief, through their Recreational Committees. These pleasures are suited to the individual needs of every type of patient. They include film shows, high class vaudeville entertainments, concerts, educational lectures and such games and sports as may be indulged in by recovering patients. An important feature of this department is the Bureau of Musical Activities, and many have been the donations of band instruments, music and offers of service from music houses, musicians and teachers, who are lending their help in gratifying the desire not only to hear, but to create good music, that lies in the hearts of our boys. One of the greatest aids to this service has been in the donation by different teachers of a definite number of hours each week to provide instruction in the hospitals.

And so, to the question, "Now that the war is done, is the Red Cross work over?" we answer, "No, not only is it not over, but what has been done is but a beginning." With past experience to build on and the future needs so plain to our sight, we point down the long road ahead and say with hope, confidence and the joy of service, *"The Work of the Red Cross Goes On."*

BACK TO THE LAND.—The Home Service Section of the American Red Cross will continue, not only through the period of demobilization, but also as a peace time activity, and in some communities it will help others besides the families of soldiers and sailors. This has been decided at National Headquarters after a recent conference of directors of civilian relief work in the various divisions and in response to urgent requests which came from all over the United States. This desire for the expansion of Home Service came particularly from communities where there are no other organized agencies for social service.

